# Suppression of Gluconeogenic Gene Expression by LSD1-Mediated Histone Demethylation

**DOI:** 10.1371/journal.pone.0066294

**Published:** 2013-06-05

**Authors:** Dongning Pan, Chunxiao Mao, Yong-Xu Wang

**Affiliations:** Program in Gene Function and Expression and Program in Molecular Medicine, University of Massachusetts Medical School, Worcester, Massachusetts, United States of America; Albany Medical College, United States of America

## Abstract

Aberrant gluconeogenic gene expression is associated with diabetes, glycogen storage disease, and liver cancer. However, little is known how these genes are regulated at the chromatin level. In this study, we investigated in HepG2 cells whether histone demethylation is a potential mechanism. We found that knockdown or pharmacological inhibition of histone demethylase LSD1 causes remarkable transcription activation of two gluconeogenic genes, FBP1 and G6Pase, and consequently leads to increased de novo glucose synthesis and decreased intracellular glycogen content. Mechanistically, LSD1 occupies the promoters of FBP1 and G6Pase, and modulates their H3K4 dimethylation levels. Thus, our work identifies an epigenetic pathway directly governing gluconeogenic gene expression, which might have important implications in metabolic physiology and diseases.

## Introduction

Hepatic glucose production is essential for maintaining blood glucose homeostasis, which ensures a steady fuel supply for many cell types in the body. Hepatic glucose production is accomplished by two processes, initially via glycogenolysis that breaks down glycogen in the first few hours after a meal, and subsequently via gluconeogenesis that de novo synthesizes glucose from non-carbohydrate precursors during prolonged fasting. Uncontrolled gluconeogenesis is a major contributor to hyperglycemia in both type 1 and type 2 diabetes [1], [2], [3]. While gluconeogenesis runs in the opposite direction of glycolysis and shares several reverse enzymatic reactions with glycolysis, three steps, catalyzed by a separate set of key enzymes, phosphoenolpyruvate carboxykinase (PEPCK), fructose-1,6-bisphosphatase (FBP1) and glucose 6-phosphatase (G6Pase), are non-reversible and largely determine the rate of gluconeogenesis. G6Pase additionally catalyzes the terminal step in the glycogenolytic pathway. Deficiency in G6Pase in patients results in glycogen storage disease Ia (GSD Ia), exhibiting hypoglycemia and abnormal hepatic accumulation of glycogen [4].

These key gluconeogenic enzymes are also suggested to play potentially important roles in suppression of liver carcinogenesis. Dramatic decrease of PEPCK, FBP1 and G6Pase gene expression was observed in hepatocellular carcinoma (HCC) developed in a mouse model and in the majority of primary human HCCs [5]. A separate study showed that FBP1 promoter is hypermethylated at the CpG sites in primary human HCCs and HCC cell lines, which results in their lower FBP1 expression relative to normal cells [6]. Importantly, restoration of FBP1 expression inhibits cancer cell proliferation [6]. In patients with G6Pase mutations (GSD1a), hepatocellular adenomas are the most severe complication with a 75% prevalence; 10% of them eventually transform to HCC [7], [8], [9], [10]. Similarly, liver-specific G6Pase knockout mice develope hepatocellular adenomas [11]. These results indicate suppressive functions of FBP1 and G6Pase in hepatocarcinogenesis. While the underlying mechanism remains obscure, reduction of FBP1 level might lead to a higher concentration of its substrate fructose-1,6-bisphosphate, which is an allosteric activator of glycolytic enzyme pyruvate kinase. Thus, a higher concentration of fructose-1,6-bisphosphate would in turn causes increased aerobic glycolysis, a process that is essential for cancer cell growth. Alternatively, downregulation of G6Pase is likely to increase the concentration of its substrate glucose-6-phosphate, which can be used by pentose phosphate shunt pathway to produce ribose-5-phosphate for nucleotide synthesis, as has been alluded [5]. Indeed, it has been observed that transformation of hepatocytes to cancer cells is usually associated with a reduction in gluconeogenesis, an activation of the glycolytic pathway and the pentose phosphate shunt pathway [12].

Given the roles of these key gluconeogenic enzymes in diabetes, glycogen storage disease and tumorigenesis, their transcriptional regulation have been explored extensively. These studies identified a number of transcription factors (e.g., CREB, FOXO1, FOXA2, C/EBPs, HNF4α, GR, Nur77, and RORα) and co-activators (e.g., PGC-1α, CRTC2, SIRT1, p300/CBP, SRC-1, and SRC-2) [13], [14] that assemble on the well-defined PEPCK and G6Pase promoters [15], [16], [17], [18] to drive their gene expression. Chromatin regulation is another fundamental process controlling gene expression. One study reported that the level of histone H3 arginine 17 dimethylation at the PEPCK promoter correlates with PEPCK gene expression [19]. We recently showed that histone demethylase Jhdm1a indirectly suppresses the expression of PEPCK and G6Pase through demethylation of H3 lysine 36 (H3K36) at the C/EBPα locus [20]. Clearly, our current understanding of gluconeogenic gene expression at the chromatin level is very rudimentary.

LSD1 is the first histone demethylase identified [21]. LSD1 associates with CoREST, CtBP and NuRD co-repressor complexes [22], [23], [24] and demethylates monomethyl- and dimethyl-H3 lysine 4 (H3K4) of its target genes [21], thereby leading to transcriptional repression. LSD1 was shown to function in diverse processes such as stem cell renewal and differentiation, embryonic and organ development, and carcinogenesis [24], [25], [26], [27], [28], [29], [30]. Here, we identify LSD1 as a key negative regulator of FBP1 and G6Pase expression and reveal a direct epigenetic mechanism underlying gluconeogenesis.

## Results

### Knockdown of LSD1 promotes FBP1 and G6Pase expression in human HepG2 cells and in primary mouse hepatocytes

**Figure 1:**
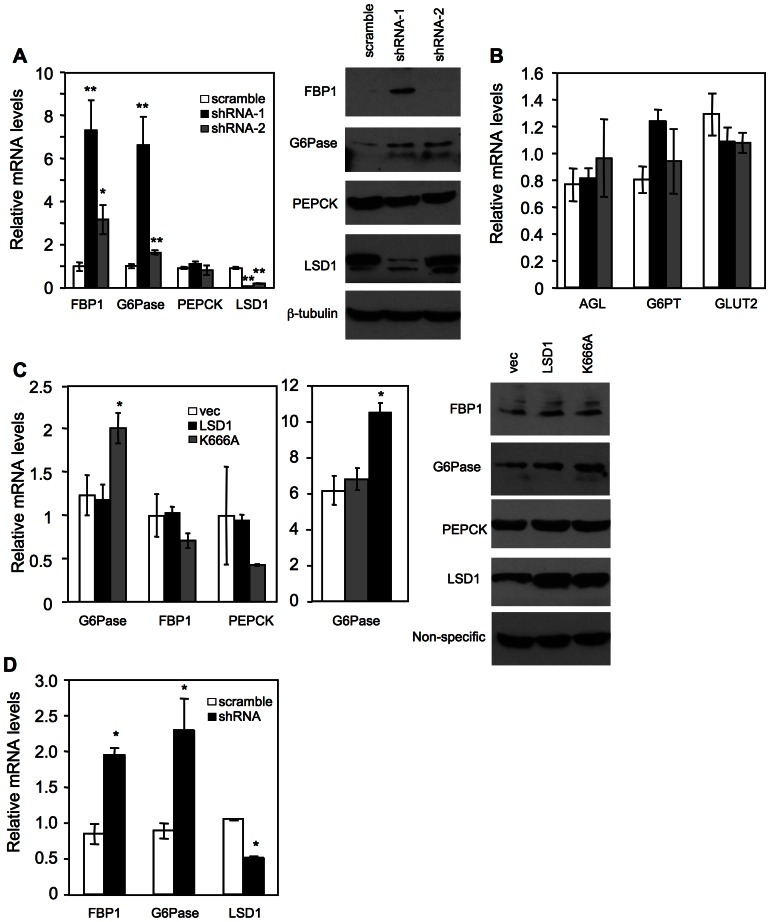
LSD1 regulates FBP1 and G6Pase expression in cultured hepatic cells. (**A, B**) LSD1 was knocked down in HepG2 cells with shRNA lentiviruses. Gene expression (n = 3–4) was determined with quantitative real-time PCR. Full names of individual genes and QPCR primers used are listed in Table 1. Right panel in (**A**), protein levels analyzed by western blot. (**C**) Lentiviral overexpression of LSD1 point mutant (K666A) increases G6Pase expression in HepG2 cells. Middle panel, G6Pase levels in HepG2 cells treated with a combination of dibutyryl cyclic-AMP (cAMP, 0.5 mM) and dexamethasone (Dex, 1 µM) for 6hr. Right panel, protein levels analyzed by western blot. Experiments were repeated three times with similar results. (**D**) Gene expression in mouse primary hepatocytes with LSD1 knockdown (n = 2). *, P<0.05; **, P<0.01.

As discussed above, abnormal gluconeogenic gene expression not only causes metabolic diseases but also contributes to hepatocarcinogenesis. We investigated whether these genes are controlled epigenetically. We performed our studies in HepG2 cells, as many known transcriptional pathways regulating gluconeogenic gene expression are retained in this HCC cell line. We generated two human LSD1 shRNA knockdown constructs and packaged them into lentiviruses. We infected HepG2 cells with the knockdown and scramble control viruses at a similar number of viral particles. We found that both LSD1 knockdown constructs significantly promoted the expression of FBP1 and G6Pase mRNA that were correlated with the LSD1 knockdown efficiency (Figure 1A). We typically observed a more than 5-fold induction of FBP1 and G6Pase expression in the first knockdown construct, which resulted in an increase of FBP1 and G6Pase protein levels. Interestingly, PEPCK expression was not affected by LSD1 knockdown (Figure 1A). This is in contrast to our previous findings that knockdown of histone demethylase Jhdm1a increased the expression of PEPCK and G6Pase but not FBP1 [20]. Thus, gluconeogenic genes can be differentially regulated by multiple histone demethylases.

**Table 1:** Gene full names and primersequences used in this study.

	Gene full name	Sequences for shRNA, QPCR and CHIP assays
		shRNA target sequence
LSD1	lysine (K)-specific demethylase 1A	GCCTAGACATTAAACTGAATA (human, construct 1)GCTCCAATACTGTTGGCACTA (human, construct 2)GGATGGGATTTGGCAACCTT (mouse)
		Primers used for real-time QPCR
LSD1	lysine (K)-specific demethylase 1A	F: TCCCTTAAGCACTGGGATCA (human)R: CCACAGGCACACACGAGTAG (human)F: GCCTGTTTCCCAGACATCAT (mouse)R: CAGCTGGATCTTTGGGTTGT (mouse)
FBP1	fructose-1,6-bisphosphatase 1	F: CCCCAGATAATTCAGCTCCTTA (human)R: GTTGCATTCGTACAGCAGTCTC (human)F: GGATTGTGGTGTCAACTGCTT (mouse)R: AGTCCTTGGCATAACCCTCAT (mouse)
G6Pase	glucose-6-phosphatase, catalytic subunit	F: GGGAAAGATAAAGCCGACCTAC (human)R: CAGCAAGGTAGATTCGTGACAG (human)F: AAGCCAACGTATGGATTCCG (mouse)R: ACAGCAATGCCTGACAAGACT (mouse)
PEPCK	phosphoenolpyruvate carboxykinase 1 (soluble)	F: TGACAACTGCTGGTTGGCTR: TGGTGCGACCTTTCATGC
AGL	amylo-alpha-1, 6-glucosidase, 4-alpha-glucanotransferase	F: GGATGGGTAATGGGAGATGAR: TAACACTGTCTCCCCAGCAA
G6PT	solute carrier family 37 (glucose-6-phosphate transporter), member 4	F: CCATGTACCTCTTCCGGGTAR: GCCATACGAGGAGAAACCAA
GLUT2	solute carrier family 2 (facilitated glucose transporter), member 2	F: ATGTGGCTCAGCAATTTTCCR: CCAACTCCAATGGTTGCATA
NUR77	nuclear receptor subfamily4, group A, member 1	F: CACAGCTTGCTTGTCGATGTR: GGTTCTGCAGCTCCTCCA
NOR1	nuclear receptor subfamily4, group A, member 3	F: CACCTTCTCCTCCAATCTGCR: GCCTGGTCAGTGGGACAGTA
SRC-2	nuclear receptor coactivator 2	F: GCACAAACGAAGAGCAAACTCR: CCATCGTTTGTCCAGTCAGAT
PGC-1α	peroxisome proliferator-activated receptor gamma, coactivator 1 alpha	F: CACAGTCGCAGTCACAACACTR: TTCCACACTTAAGGTGCGTTC
C/EBPα	CCAAT/enhancer binding protein (C/EBP), alpha	F: TGGACAAGAACAGCAACGAGTAR: ATTGTCACTGGTCAGCTCCAG
β-actin	actin, beta	F: CCTGGCACCCAGCACAATR: GCCGATCCACACACGGAGTACT
U36B4	ribosomal protein, large, P0	F: AGATGCAGCAGATCCGCAR: GTTCTTGCCCATCAGCACC
		CHIP assays primers
FBP1	promoter −1293∼−1166promoter −214∼−119	F: TCTCCAGTGGGTTCCTCCTAR: ACCGGTGGAGCTGAAATAGAF: CAGCCCAGGAAGACTAGGGR: GCCTGCTTGGATCTTCAGAC
G6Pase	promoter −242∼−132promoter −150∼−58	F: CCAAGAAGCATGCCAAAGTTR: TGCAAACATGTTCAGGGTGAF: CACCCTGAACATGTTTGCATR: AGCCCTGATCTTTGGACTCA
GAPDH	5′UTR 443−631	F: CGGCTACTAGCGGTTTTACGR: AAGAAGATGCGGCTGACTGT

Because G6Pase also catalyzes the terminal step of glycogenolysis, we examine whether LSD1 regulates the expression of additional enzymes in this pathway. Knockdown of LSD1 had no effect on the expression of glycogen debranching enzyme (amylo-alpha-1, 6-glucosidase, 4-alpha-glucanotransferase, AGL) and glucose-6-phosphate transporter (G6PT) (Figure 1B). The expression of Glut2, which transport glucose out of hepatocytes, was also not changed. Thus, LSD1 regulates only a subset of genes in the gluconeogenic and glycogenolytic pathways.

We next carried out LSD1 overexpression experiments via lentiviral infection. Overexpression of wild type LSD1 did not affect the expression of G6Pase in HepG2 cells, indicating that endogenous LSD1 level is not a limiting factor. Indeed, endogenous LSD1 protein was abundant and readily detectable in these cells by western blotting analysis (Figure 1A). On the other hand, overexpression of a LSD1 point mutant (K666A), which abolishes its demethylation activity [27], [31], leads to a reproducible increase of G6Pase expression (Figure 1C), indicating a dominant negative effect, as would be expected. These data provide further evidence for the role of LSD1 in suppression of gluconeogenic gene and indicate a requirement of its demethylation activity.

We addressed whether regulation of gluconeogenic gene expression occurs in normal hepatocytes. We infected mouse primary hepatocytes with lentiviruses expressing a mouse LSD1 knockdown construct. Despite that LSD1 was only knocked down by 50%, a derepression of FBP1 and G6Pase expression was observed (Figure 1D). Therefore, LSD1-mediated suppression of gluconeogenic gene expression is not limited to liver cancer cells, and might have a physiological relevance.

### Pharmacological inhibition of LSD1 phenocopies the effects of LSD1 knockdown on gluconeogenic gene expression

**Figure 2:**
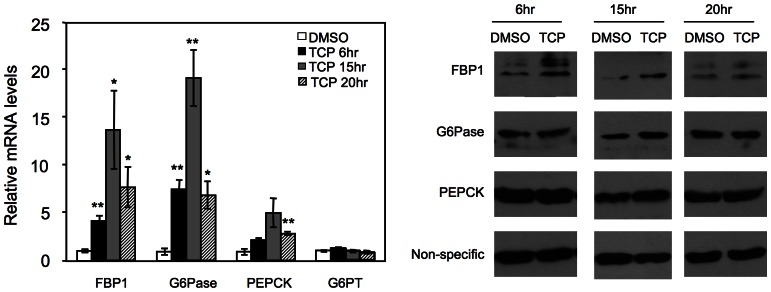
Induction of gluconeogenic gene expression by LSD1 inhibitor TCP. HepG2 cells were treated with 1 mM TCP or DMSO. Total RNA was extracted at indicated time and gene expression was examined with QPCR, n = 3. Right panel, protein levels at indicated time were analyzed by western blot.

Tranylcypromine (TCP) potentially inhibits the demethylation activity of LSD1 [32], [33]. We treated HepG2 cells with TCP and analyzed gene expression. FBP1 and G6Pase, but not G6PT, were robustly induced by TCP treatment at all three indicated time points (Figure 2). The rapid response to TCP treatment suggests that FBP1 and G6Pase might be direct target genes of LSD1. PEPCK was slightly increased during prolonged TCP treatment; this increase is probably indirect, as it was not seen in either LSD1 knockdown or 6 hr TCP treatment. Thus, the LSD1 inhibitor recapitulates the role of LSD1 knockdown for gluconeogenic gene expression.

### LSD1 regulates glucose production in vitro

**Figure 3:**
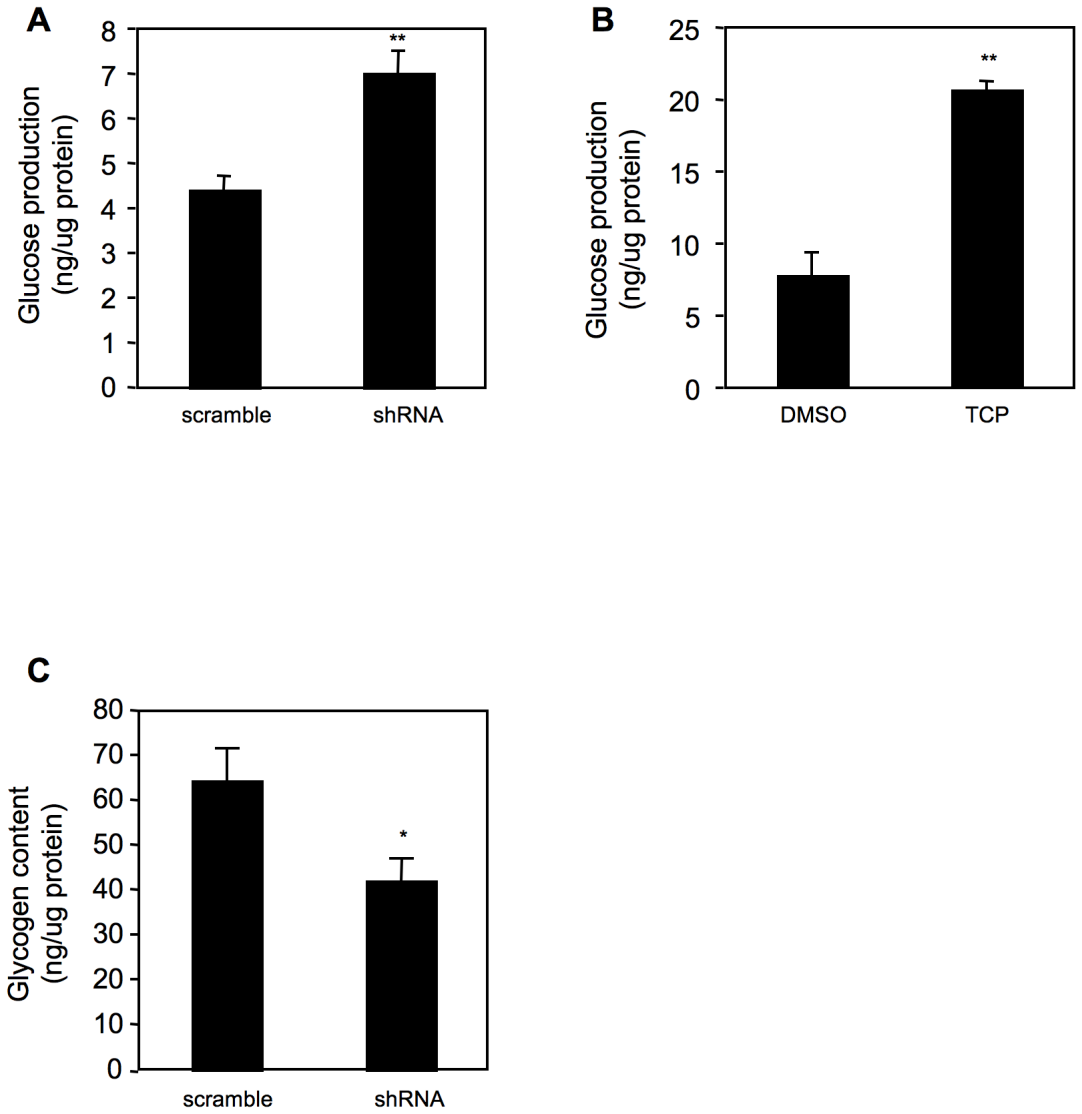
LSD1 regulates glucose production in HepG2 cells. Assays were performed as described in Materials and Methods. (**A, B**) gluconeogenesis in LSD1 knockdown cells (**A**) or in cells pre-treated with TCP for 16 hr (**B**), n = 4. (**C**) glycogen content in LSD1 knockdown cells, n = 3. *, P<0.05; **, P<0.01.

To determine whether suppression of gluconeogenic gene expression by LSD1 has a functional consequence, we cultured LSD1 knockdown HepG2 cells in a medium that contains gluconeogenic substrates for 6 hr and measured de novo glucose synthesis. As shown in Figure 3A, knockdown of LSD1 significantly promoted glucose synthesis. Similar results were obtained when cells were treated with the LSD1 inhibitor TCP (Figure 3B). Because G6Pase also catalyzes the terminal step of glycogenolysis, we suspect that increased expression of G6Pase by LSD1 knockdown might result in diminished intracellular glycogen level. To test this, we first cultured HepG2 cells in a high glucose medium to allow glycogen accumulation. Cells were then shifted to a medium without glucose for 4 hr. Intracellular glycogen was isolated and assayed. We found that glycogen content was significant lower in LSD1 knockdown cells (Figure 3C). Our data suggest that LSD1 negatively regulates both gluconeogenesis and glycogenolysis, at least in vitro.

### LSD1 associates with gluconeogenic gene promoters

**Figure 4:**
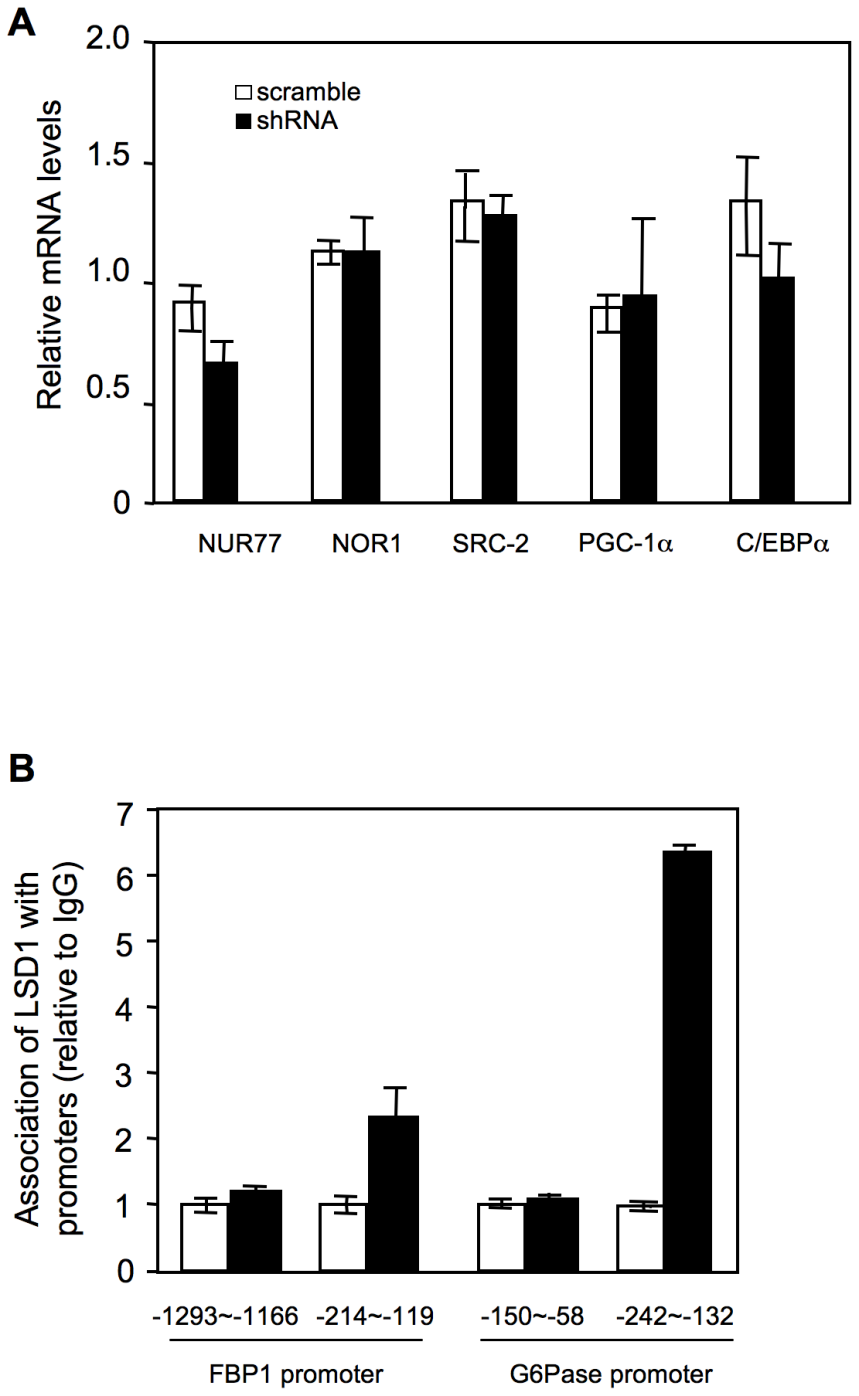
LSD1 associates with FBP1 and G6Pase promoters. (**A**) LSD1 was knocked down in HepG2 cells with shRNA lentiviruses. Expression levels of gluconeogenic regulators were examined, n = 3−4. (**B**) LSD1 associates with FBP1 and G6Pase promoters. Data were shown from one representative of two independent experiments with similar results.

We investigated how LSD1 represses gluconeogenic gene expression. Previous work by others has shown that Nur77, Nor1 and transcriptional co-activator Src-2 upregulate G6Pase but not PEPCK expression [34], [35], similar to what we observed for LSD1 knockdown. However, knockdown of LSD1 had no effect on the levels of Nur77, Nor1 and Src-2 (Figure 4A). We also did not observe an increase of other transcriptional regulators important for both PEPCK and G6Pase expression, such as PGC-1α and C/EBPα. These results, together with the rapid induction by TCP, raise the possibility that LSD1 directly represses FBP1 and G6Pase expression. To test this idea, we performed chromatin immunoprecipitation (ChIP) assays in HepG2 cells to determine whether endogenous LSD1 protein is located at their promoters. As shown in Figure 4B, LSD1 was detected at both FBP1 promoter region (−214 to −119 bp) and G6Pase promoter region (−242 to −131 bp). These regions have been shown to interact with several transcription factors [6], [17], [18], [36], [37]. Thus, LSD1 occupies the FBP1 and G6Pase promoters, likely through its interaction with transcription factors.

### Both knockdown and inhibition of LSD1 increase the levels of H3K4me2 at gluconeogenic gene promoters

**Figure 5:**
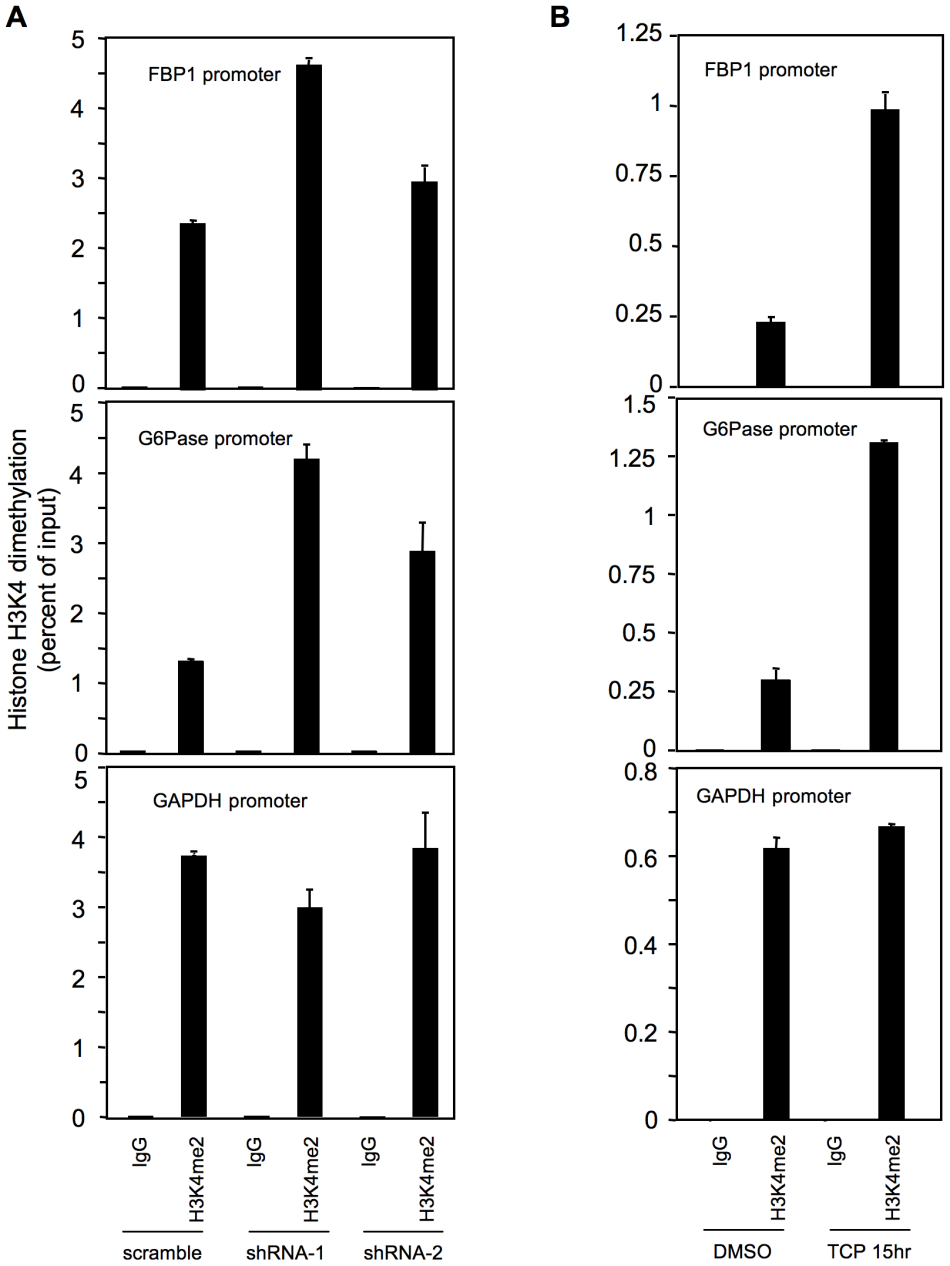
Regulation of H3K4me2 at FBP1 and G6Pase promoters by LSD1. HepG2 cells were infected with LSD1 knockdown lentiviruses (**A**) or treated with TCP (1 mM) (**B**), and H3K4me2 levels were examined at FBP1, G6Pase and GAPDH promoters. All data were shown from one representative of two independent experiments with similar results.

H3K4 dimethylation (H3K4me2) peaks around transcriptional start sites and its level positively correlates with gene expression [38]. Given the association of LSD1 with the FBP1 and G6Pase promoters, we performed ChIP assays to examine whether LSD1 regulates the dynamics of H3K4 methylation in HepG2 cells. H3K4 at both FBP1 and G6Pase promoters are dimethylated (Figure 5A and B), consistent with the idea that the two genes are active or poised for activation in HepG2 cells. Knockdown of LSD1 elevated the levels of H3K4me2 at the two promoters (Figure 5A). TCP treatment produced a similar effect (Figure 5B). These results support a model that LSD1 directly demethylates H3K4me2 at FBP1 and G6Pase promoters and that induction of FBP1 and G6Pase expression by LSD1 knockdown or inhibition is due to an increase of H3K4me2 levels.

## Discussion

FBP1 and G6Pase genes play important roles in gluconeogenesis, glycogen mobilization, and liver tumorigenesis. Their expression levels are tightly controlled by an array of transcription factors and co-factors [13], [14]; however, little is known how they are regulated by the chromatin landscape. Here, we identify LSD1 as a key epigenetic regulator that directly modulates the expression of FBP1 and G6Pase. Knockdown or pharmacological inhibition of LSD1 causes an increase of H3K4me2 levels at FBP1 and G6Pase promoters and an upregulation of their expression, resulting in enhanced glucose synthesis and diminished intracellular glycogen accumulation. Our data suggest that the dynamic H3K4me2 marker is important for proper gluconeogenic gene expression and that LSD1-mediated demethylation is a potentially important epigenetic mechanism underlying the gluconeogenic process. However, it should be noted that this mechanism was revealed through in vitro cell culture studies and its relevance to in vivo physiology and pathophysiology remains to be validated with animal models. If our findings can be extended to in vivo studies, it raises a possibility for a potential utility of LSD1 inhibitors in the treatment of a variety of forms of G6Pase-independent glycogen storage diseases and hepatocellular carcinoma.

In contrast to H3K36 demethylase Jhdm1a, which indirectly regulates PEPCK and G6Pase expression through demethylation of C/EBPα locus [20], several lines of evidence support a direct role of LSD1 for the suppression of FBP1 and G6Pase expression. First, we found no increase of mRNA levels of known gluconeogenic transcriptional regulators upon LSD1 knockdown, although we can not rule out the possibility that any of the factors might be a non-histone substrate for LSD1; second, induction of FBP1 and G6Pase in response to TCP treatment is very rapid; third, LSD1 occupies FBP1 and G6Pase promoters and knockdown or inhibition of LSD1 leads to an increase of H3K4me2 levels at these promoters. However, the detailed mechanism of how LSD1 is recruited to these promoters and represses their transcription remains to be determined. Interestingly, LSD1 is located at a NF-κB-binding site on the FBP1 promoter. Studies have shown that activation of NF-κB results in DNA methylation of this promoter and consequently FBP1 expression silencing [6], [37]. As demethylation of H3K4 appears to be a prerequiste for DNA methylation [39], one testable model is that LSD1 is recruited to the FBP1 promoter by NF-κB, and then elicits DNA hypermethylation through removal of H3K4me2. This model might also involve the NR4A orphan nuclear receptor members. NR4A members regulate FBP1 and G6Pase expression, but not PEPCK expression [34]. NR4A members have also been shown to recruit LSD1-CoREST complex to NF-κB target gene promoters to suppress their expression [40]. Thus, these studies indicate a possible molecular connection among LSD1, NF-κB and NR4A members that governs FBP1 and G6Pase gene expression. Further studies are necessary to elucidate whether and how LSD1 coordinates with these factors and other epigenetic modifiers in the gluconeogenic process.

Our studies raise several mechanistic questions that warrant investigation. For example, what is the opposing histone methyltransferase(s) that methylates H3K4 at the FBP1 and G6Pase promoters? What other components in the LSD1-associated co-repressor complexes are required for gluconeogenic gene repression? How is LSD1-mediated demethylation at the FBP1 and G6Pase promoters regulated in response to physiological cues and disease states? Answering these questions in the future will provide important insights into the epigenetic control of glucose metabolism and its impacts in diseases.

## Materials and Methods

### Cell cultures

HepG2 cells (ATCC, # HB-8065) were grown in DMEM medium (Invitrogen #11965-092) with 10% fetal bovine serum (Atlanta Biologicals #s11150), 100 U/ml penicillin and 10 µg/ml streptomycin. Primary mouse hepatocytes were prepared and cultured as described previously [20], [41].

### Lentiviral knockdown

LSD1 knockdown or scramble oligonucleotides were inserted into pSP108 lentiviral vector (Addgene). Lentiviral particles were produced in 293T cells. HepG2 cells in 6-well plates were infected with a similar number of viral particles. Cells were transferred into 10-cm culture dishes next day and selected with 2 µg/ml puromycin for three days. Cells were then re-plated into 12-well plates at a similar confluency and cultured in the presence of puromycin for two more days.

### Lentiviral overexpression

Mouse wild-type and point mutant LSD1 coding sequence were cloned into pENTR-1A vector and recombined with plenti-CMV/neo plasmid to generate lentiviral constructs. The lentivirus was generated as described previously [20]. HepG2 cells were transduced with similar number of viral particles, selected with 2 mg/ml G418 and cultured as above.

### TCP treatment

HepG2 cells were plated into 6-well plates. At 80% confluency, cells were treated with DMSO or 1 mM trans-2-phenylcyclopropylamine hydrochloride (TCP, sigma #P8511). mRNA or protein levels were determined.

### Gluconeogenic assay

Cells were treated with 0.5 mM dibytyryl cyclic-AMP (cAMP) and 1 µM dexamethasone (Dex, 1μM) for 5 hr. Cells were then washed 3 times with PBS and incubated in glucose/phenol-red free DMEM medium containing 2 mM sodium pyruvate and 20 mM sodium lactate for 6 hr. Glucose levels in the medium were measured with an Amplex red glucose assay kit (Invitrogen, #A22189).

### Glycogen content

HepG2 cells were incubated with high glucose (50 mM) DMEM medium for 2 hr, and then shifted to glucose-free DMEM medium for 4 hr. The glycogen contents in the cells were measured as published with minor modification [42]. Briefly, after washed 3 times with PBS, the cells were collected in 50 mM sodium acetate (pH4.5) and lysed by sonication on ice. The supernatants of the disruptants were incubated at 55°C for 1hr in the absence or presence of amyloglucosidase (200 mU/ml, sigma #A7420). After the reaction, the samples were deproteinized, neutralized and the glucose levels were measured with an Amplex red glucose assay kit. The glycogen contents were calculated by subtracting the glucose levels of the undigested samples from those of the digested samples.

### Western Blotting

Cells were collected in lysis buffer [100 mM NaCl, 50 mM Tris (pH 7.5), 0.5% Triton X-100, 5% (w/v) glycerol] with protease inhibitor tablets (Roche) and PMSF, and incubated on ice for 30 min. The lysates were cleared by centrifugation at 16,000 g for 10 min at 4°C. Equal amounts of protein sample were then separated on 8% SDS polyacrylamide gels. Antibodies against LSD1 (Abcam#ab17721), FBP1 (Abgent #AP7385C), G6Pase (Santa Cruz #sc-25840), PEPCK (ABcam #ab28455) and β-tubulin (DSHB #E7-S) were used.

### RNA isolation and real time quantitative PCR

Total RNA was isolated using Trizol (Invitrogen) following manufacturer’s instructions and was subjected to reverse transcription with SuperScript III Reverse Transcriptase (Invitrogen #18080-044). Real time quantitative PCR was performed using reagent iTaq SYBR Green Supermix with ROX (Bio-rad #172-5850). All the signals were normalized with β-actin or U36B4 and the 2^−ΔΔC^_T_ method was used for quantification.

### Chromatin immunoprecipitation assays

HepG2 cells on 10-cm dishes (1×10^7^ cells/dish) were fixed in 1% formaldehyde for 5 min at room temperature. Cells were washed with cold PBS containing protease inhibitor cocktail (Roche) and pelleted by centrifugation. The cell pellets were suspended in ChIP lysis buffer (50 mM Tris-HCl pH 8.1, 10 mM EDTA, 1% SDS and protease inhibitors). Fragmentation of the DNA (100–200bp) was achieved by a 50 sec sonication (10 sec pulse on and 5 sec pulse off) on ice with ultrasonic processor (Cole Parmer) at 40% amplitude. The sheared chromatin was cleared by centrifugation at 16,000 g for 10 min at 4°C and diluted 10-fold in CHIP IP buffer (16.7 mM Tris-HCl pH 8.1, 1.2 mM EDTA, 1.1% Triton X-100, 0.01% SDS, 167 mM NaCl). 50% protein A-sepharose slurry (GE Healthcare #17-0780-01) was added to the sample and incubated for 1 hr at 4°C with rotation. The pre-cleared chromatin samples were then immunoprecipitated with antibodies against LSD1 (5 µg, Abcam #ab17721), dimethyl-H3K4 (5 µg, Millipore #07-030) or normal rabbit IgG (Sigma #I-5006). Two percent of the chromatin samples were saved as input control. DNA samples from the CHIP were purified with PCR purification kits (Qiagen) and applied for quantification by real time PCR. CHIP signal was normalized with input signal.

### Statistical analysis

Student’s t test (two-tailed) was used for statistical analysis. P<0.05 was considered significant. Data are presented as mean ± s.e.m.
